# Minocycline alters behavior, microglia and the gut microbiome in a trait-anxiety-dependent manner

**DOI:** 10.1038/s41398-019-0556-9

**Published:** 2019-09-13

**Authors:** Anna K. Schmidtner, David A. Slattery, Joachim Gläsner, Andreas Hiergeist, Katharina Gryksa, Victoria A. Malik, Julian Hellmann-Regen, Isabella Heuser, Thomas C. Baghai, André Gessner, Rainer Rupprecht, Barbara Di Benedetto, Inga D. Neumann

**Affiliations:** 1 University of Regensburg, Department of Behavioural and Molecular Neurobiology, Regensburg Center of Neuroscience, Regensburg, Germany; 2 University Hospital Frankfurt, Goethe University, Department of Psychiatry, Psychosomatic Medicine and Psychotherapy, Frankfurt, Germany; 3 University Hospital Regensburg, Institute of Clinical Microbiology and Hygiene, Regensburg, Germany; 4 University Regensburg, Department of Psychiatry and Psychotherapy, Regensburg Center of Neuroscience, Regensburg, Germany; 5 Department of Psychiatry, Charité-Campus Benjamin Franklin, Section Neurobiology, University Medicine Berlin, Berlin, Germany

**Keywords:** Neuroscience, Pharmacology, Depression

## Abstract

Major depressive disorder is the main cause of disability worldwide with imperfect treatment options. However, novel therapeutic approaches are currently discussed, from augmentation strategies to novel treatments targeting the immune system or the microbiome-gut-brain axis. Therefore, we examined the potential beneficial effects of minocycline, a tetracycline antibiotic with pleiotropic, immunomodulatory action, alone or as augmentation of escitalopram on behavior, prefrontal microglial density, and the gut microbiome in rats selectively bred for high anxiety-like behavior (HAB). We show that concomitant with their high innate anxiety and depression, HABs have lower microglial numbers in the infralimbic and prelimbic prefrontal cortex and an altered gut microbiota composition compared with controls. Three weeks of minocycline treatment alleviated the depressive-like phenotype, further reduced microglial density, exclusively in male HAB rats, and reduced plasma concentrations of pro-inflammatory cytokines. However, coadministration of escitalopram, which had no effect alone, prevented these minocycline-induced effects. Moreover, minocycline led to a robust shift in cecal microbial composition in both HABs and rats non-selected for anxiety-like behavior. Minocycline markedly increased relative abundance of Lachnospiraceae and Clostridiales Family XIII, families known for their butyrate production, with a corresponding increase and positive correlation in plasma 3-OH-butyrate levels in a trait-dependent manner. Thus, our data suggest that the antidepressant effect of minocycline is sex- and trait-dependent, associated with a reduced microglial number in the prefrontal cortex, and with changes in microbial composition and their metabolites. These results further support the microbiome-gut–brain axis as potential target in the treatment of depression.

## Introduction

According to the World Health Organization, depression has become the leading cause of disability worldwide affecting approximately 300 million people with a lifetime prevalence of 10.7% in men and 18.1% in women in the US^1^. Numerous treatment options for major depressive disorder (MDD) are available such as the frequently prescribed and highly efficacious selective serotonin reuptake inhibitor (SSRI) escitalopram^2,3^. However, antidepressants display a therapeutic lag of 2–4 weeks, and only 70% of patients respond sufficiently^4^. To achieve therapies with faster onset, two major approaches can be utilized, i.e. optimization of existing options or the discovery of agents with novel mechanisms of action. Treatment regimens move towards augmentation strategies, a combination of conventional antidepressants with non-antidepressant substances to increase efficacy and overcome treatment resistance^5^. Regarding novel targets, central inflammation and the microbiome-gut–brain axis are two areas receiving growing traction^6,7^.

Thus, dysregulation in microglia, the immune system of the brain, as well as the gut microbiome have been associated with MDD^8–11^. A significant influence of microbiota on microglia activation and maturation, potentially via bacteria-produced short-chain fatty acids like butyrate, has been reported^12,13^. This points towards a complex role of microbiota and microglia in anxiety and depression, suggesting a subtle interplay between behavior, microglia and microbiota. In light of this, one attractive treatment option is the pleiotropic broad-spectrum tetracycline antibiotic minocycline, with neuroprotective and anti-inflammatory properties, e.g. inhibiting microglial activation^14–17^, which also affects gut microbiota^18^. Recent clinical studies have shown that minocycline can augment the clinical efficacy of antidepressants^19–21^, an effect that was proposed to be dependent on the presence of an inflammatory state^22^. These findings are supported by several rodent studies showing that minocycline promotes active stress coping^23,24^ and reverses inflammation-induced^25,26^ as well as stress-induced depressive-like behavior^27,28^. However, substantial evidence is required from appropriate animal models regarding behavioral effects of minocycline and particularly relating to its mechanism(s) of action.

One of the most heuristic approaches to develop appropriate animal models is via selective breeding for a particular trait^29^. This approach gave rise to Wistar rats with extremely high anxiety-related behavior (HAB) based on their performance on the elevated plus-maze (EPM), a phenotype repeatedly confirmed independent of sex. Moreover, HAB rats consistently show similar characteristics as observed in MDD patients including passive stress-coping, altered serotonin and neuropeptide signaling in the brain and, consequently, dysregulated responses of the hypothalamic–pituitary–adrenal (HPA) axis^30–33^. The highly anxious and depressive-like phenotype of male HAB rats has been successfully reversed by chronic citalopram or paroxetine treatment^34,35^. However, this required an 8-week treatment regimen, suggesting HAB rats as an animal model for comorbid anxiety and depression, as well as treatment-resistant depression. Amelioration of these behavioral deficits in female HAB rats remains to be studied, an approach urgently needed considering the more frequent diagnosis of MDD in women^30^.

Changes in several cortical and limbic structures like the hippocampus, amygdala and especially the prefrontal cortex (PFC) are documented in the pathophysiology of depression^36–40^. Fittingly, intra-PFC citalopram microinfusion acted as an antidepressant^41^, whereas glial loss in this region facilitated depressive-like behavior^42^. Moreover, stress- or inflammation-induced depressive-like behavior paralleled by PFC microglial activation was prevented by minocycline treatment^25,27,43^. Importantly, acute inactivation of the infralimbic cortex in HAB rats attenuated depressive-like behavior, corresponding to the behavioral consequences of deep brain stimulation of Brodmann Area 25 in MDD patients^39,44^, indicating the translational relevance of this animal model and the importance of this region.

Based on these prerequisites, we assessed the effects of chronic minocycline treatment on anxiety- and depressive-like behavior in rats in dependence on trait anxiety levels in both sexes. For translational comparison with clinical studies using minocycline as an add-on treatment, the standard SSRI escitalopram and the augmentation of minocycline with escitalopram were included. In order to approach potential mechanisms underlying the differential treatment effects, we further examined microglia density in the PFC, peripheral immune function comprising pro-inflammatory cytokines, and alterations in gut microbiota. Male and female rats non-selected for anxiety (NAB) were included to determine a trait-dependency of treatment.

## Methods and materials

### Animals

Male and female NAB rats were either purchased from Charles River (Wistar rats; Sulzfeld, Germany) or, like all male and female HAB rats, bred at the University of Regensburg. HAB rats showing <10% of time spent on the open arms of an EPM at the age of 9 weeks were selected^45^. All rats were maintained on a 12-h light–dark cycle in a temperature controlled colony (22–24 °C; 55±5% Humidity) with free access to food and tap water (vehicle) or drug solution (minocycline and/or escitalopram, see experimental design) under facility-specific husbandry conditions according to the respective EU regulations (2010/63/EU). All experimental procedures were approved by the Committee on Animal Health and Care of the local government, and performed according to the Guide for the Care and Use of Laboratory Animals of the Government of Oberpfalz and Unterfranken, the ARRIVE guidelines^46^, and recommendations from the NIH.

### Experimental design

At the age of 11–12 weeks, rats were group-housed in cages of 3–4 rats according to their experimental group (i.e. sex and trait) and randomly assigned to the treatment. Body weight and water consumption were monitored daily to calculate the respective concentrations needed for oral drug administration via drinking water and to prepare fresh solutions daily. In the first set of experiments, 40 mg/kg/day minocycline^43^, 10 mg/kg/day escitalopram^47^, or a combination of both substances with the same concentrations, while in the second set, 80 mg/kg/day minocycline alone, all dissolved in tap water, was applied for 22 days. The treatment duration was chosen based on the fact that antidepressant treatment generally needs 2–3 weeks for the onset of therapeutic efficacy^48^ and on a previous study showing an antidepressant effect of minocycline already after 7 days^28^. Minocycline hydrochloride and escitalopram oxalate were kindly provided by the Charité Berlin and Lundbeck A/S (Copenhagen, Denmark), respectively.

**Fig. 1:**
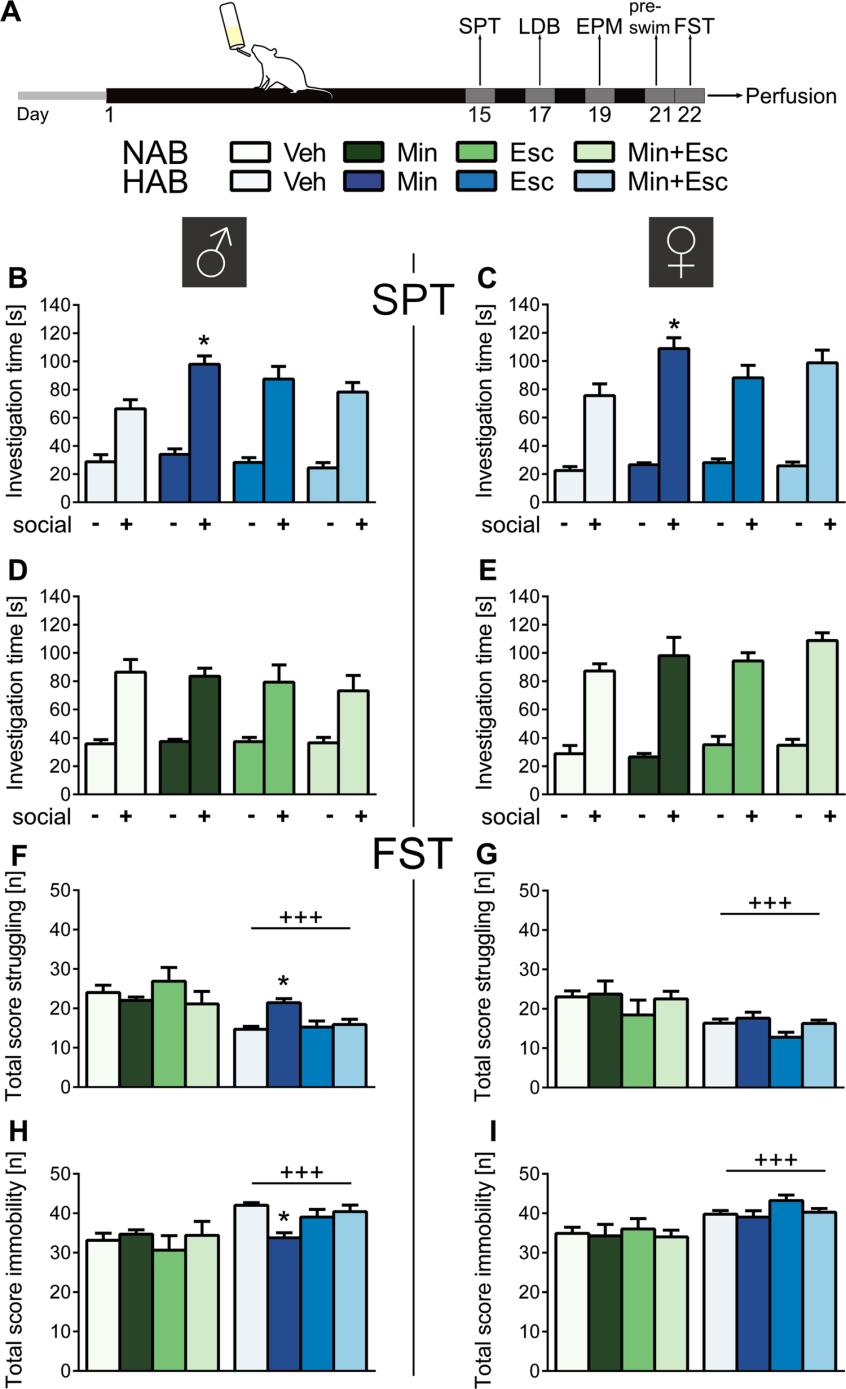
**Experimental design and behavioral outcome after treatment of male and female NAB and HAB rats with either vehicle (Veh), minocycline (Min), escitalopram (Esc), or a combination of both (Min+Esc) for 22 days**. **a** After 15 days of treatment, social preference behavior was tested in the social preference test (SPT), followed by evaluation of anxiety-like behavior in the light–dark box (LDB; day 17) and the elevated plus-maze (EPM; day 19), and of depressive-like behavior in the pre-swim (day 21) and forced swim test (FST; day 22). Social preference is reflected by longer investigation of the social (+) versus the non-social (−) stimulus in the SPT, whereas depressive-like behavior is reflected as total score spent struggling and immobile during the FST. All male (**b**, **d**) and female (**c**, **e**) HAB and NAB rats showed natural social preference (significance not indicated), which is facilitated in male and female HAB rats by Min. A depressive-like phenotype was observed in all male (**f**, **h**) and female (**g**, **i**) HAB rats in the FST compared to NAB rats except male HAB rats that were treated with Min (**f**, **h**). Data represents mean + s.e.m., *p < 0.05 vs. corresponding Veh group, +++*p* < 0.001 vs. NAB; SPT: one-way ANOVA for repeated measures; FST: two-way ANOVA, followed by a Bonferroni post hoc test

Rats were tested in the social preference test (SPT; day 15), in the light–dark box (LDB; day 17) and on the EPM (day 19), as well as in the forced swim test (FST; day 22; Fig. 1a). Behavioral tests were conducted between 0900 and 1200 except for the SPT (1900 to 2200) and behavioral analysis was conducted by an observer blind to the trait and treatment. Vaginal smears were taken after the EPM and FST to control for estrous cycle. Immediately after the FST, all rats were deeply anesthetized for transcardial perfusion (1x phosphate buffered saline and 4% paraformaldehyde), and blood, liver, fecal boli, and cecal samples were collected, snap frozen using dry ice and stored at −80 °C until further analyses. Microglia quantity was analyzed by immunofluorescent-immunohistochemistry of the microglial marker ionized calcium-binding adapter molecule-1 (Iba-1, Wako, 019-19741) on 40 µm coronal brain sections from the infralimbic and prelimbic PFC. For method details see Supplementary Methods.

### Minocycline tissue extraction and HPLC analyses

A sensitive and specific reversed-phase high-performance liquid chromatographic (HPLC) method was developed and optimized for detection of minocycline in liver tissue and fecal boli. The limit of quantitation was 50 ng/ml with an averaged recovery from homogenates of 95%, with a 5–10% intra- and inter-assay coefficient (see Supplementary Methods).

### Determination of plasma beta-hydroxybutyrate levels

Beta-hydroxybutyrate concentrations (3-OH-butyrate; stimulated by butyrate release) in plasma were determined with a commercial assay kit (Sigma-Aldrich, Germany). The enzyme reaction results in a colorimetric product proportional to the presence of the metabolite 3-OH-butyrate (see Supplementary Methods).

### Luminex® cytokine detection assay

Interferon (IFN)-γ and interleukin (IL)-12p40 concentrations were quantified in plasma using a cytokine bead immunoassay (Invitrogen/ThermoFisher Scientific, Darmstadt, Germany) and the *Luminex xMAP 100* system (Luminex, Austin, TX, USA) according to the manufacturer’s protocol (see Supplementary Methods).

### Intestinal microbiome analysis by 16S-rDNA pyrosequencing

The methods used for the isolation of DNA from stool specimens, quantification of 16S-rDNA copies by qPCR, PCR amplification of V3-V6 16S-rDNA variable regions, 454 pyrosequencing (see Supplementary Table S1 for primer sequences), sequence processing and operational taxonomic unit (OTU) clustering as well as analysis of microbial composition and global community structure are described in the Supplementary Methods.

### Statistical analysis

Behavioral data, plasma 3-OH-butyrate, and cytokine concentrations were analyzed with the software package SPSS (version 12) using either one-way analysis of variance (ANOVA; factor treatment), two-way ANOVA (factors trait×treatment) or two-way ANOVA for repeated measures (factors trait×treatment×stimulus) followed by a Bonferroni post hoc test for multiple comparisons when appropriate. For analysis of cytokine concentrations, a Mann–Whitney *U*-test was applied (for detailed statistical analysis see Supplementary Table S2 as well as figure descriptions; for detailed group sizes see Supplementary Table S2). Group sizes were defined by power analysis and experimental experience. Animals were excluded from statistical analysis, if the respective parameter differed more than twice the standard deviation from the group mean. For microbiome data, the observed OTU numbers, 16S-rDNA copy numbers, and relative abundances of bacterial taxa were analyzed in R by ANOVA with a subsequent Tukey’s test. Pairwise multilevel comparisons of Bray–Curtis distances as depicted in the PCoA plot were analyzed using the vegan package in R followed by a Bonferroni correction to adjust *p* values. Correlations between bacterial families and plasma butyrate were calculated using Spearman’s correlation coefficients in GraphPad Prism (version 5). Significance was accepted at *p* ≤ 0.05.

## Results

### Behavioral effects of minocycline in the SPT, LDB, EPM, and FST

Male and female HAB and NAB rats were treated with either minocycline (40 mg/kg), escitalopram or a combination of both via the drinking water for 22 days, and fluid intake was monitored daily (see supplementary results Table S3 and Fig. S1). In the SPT, all rats displayed natural social preference as indicated by a higher investigation time of the social versus the non-social stimulus (*p* < 0.001; Fig. 1b–e). Post hoc analyses revealed that minocycline alone facilitated social approach in male (Fig. 1b) and female (Fig. 1c) HAB rats (*p* < 0.05 vs. Veh). In both the LDB and EPM, the expected anxious phenotype of HAB rats was visible in both sexes compared to NAB rats (*p* < 0.05; Fig. S2). A treatment effect was only seen in male NAB rats, where escitalopram alone increased the time in the light box (*p* < 0.05).

Concomitant with the high anxiety phenotype, male and female HAB rats displayed reduced struggling and increased immobility in the FST compared to NABs (*p* < 0.001; Fig. 1f–i). Minocycline was the only treatment that reversed this phenotype, but exclusively in male HAB rats indicated by decreased immobility and increased struggling (*p* < 0.05 vs. Veh, Fig. 1f, h). No treatment-induced changes in locomotion in HAB rats (Fig. S2) and no influence of the female cycle on behavior was detectable (data not shown).

To assess potential sex-dependent differences in effective doses, 80 mg/kg minocycline was also administered to male and female HAB rats. Again, minocycline increased social preference in both sexes (*p* < 0.001; Fig. S3A, B) and ameliorated depressive-like behavior in male HAB rats (*p* < 0.05; Fig. S3G–J), whereas anxiety-like behavior remained unchanged (Fig. S3C–F). Hence, in all following analysis, only samples from the 40 mg/kg groups were analyzed.

### Effects of minocycline on microglial numbers in the PFC and cytokine concentrations in plasma

**Fig. 2:**
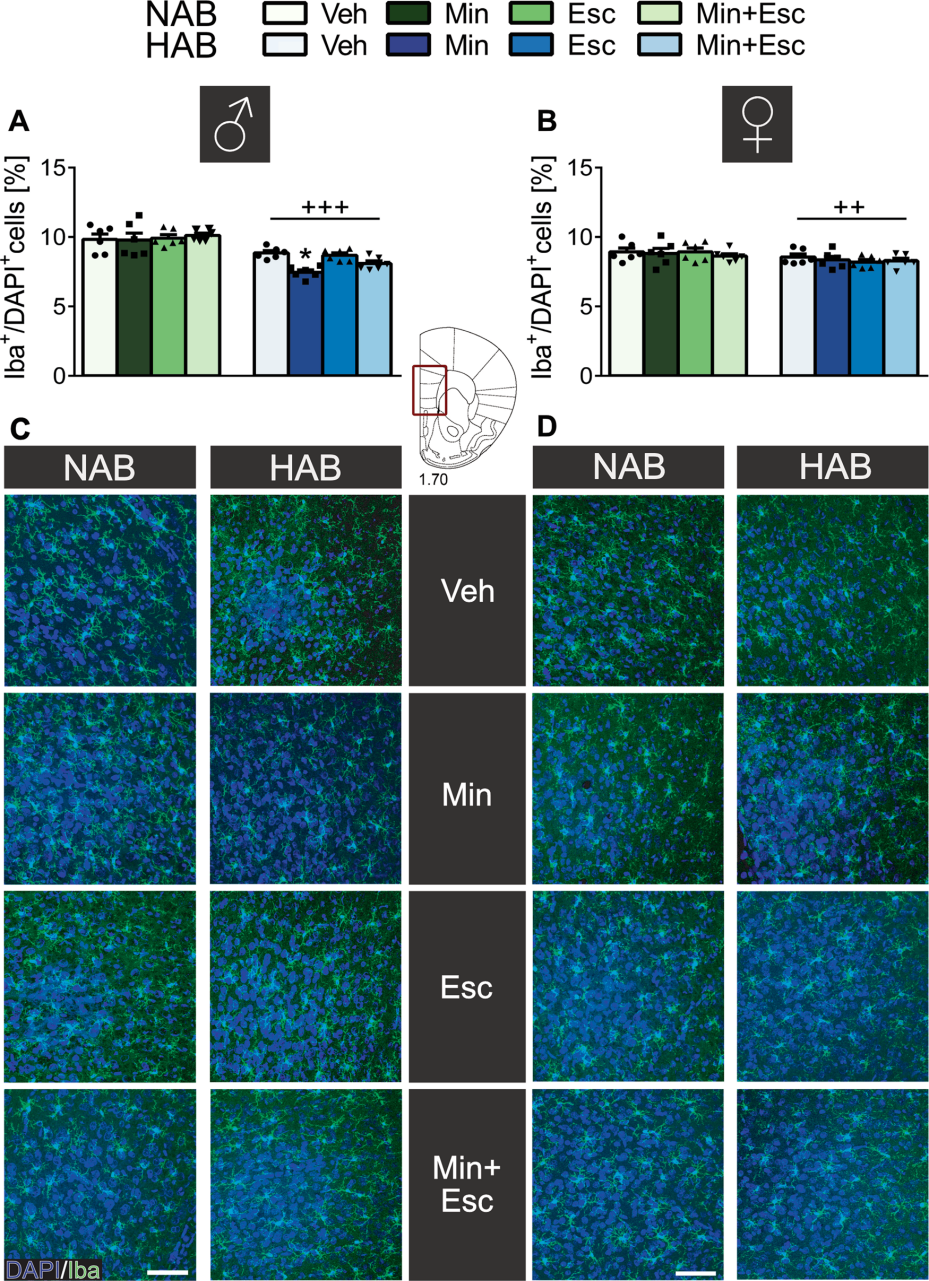
**Number of Iba-1 positive (Iba+) microglia cells in the prefrontal cortex (PFC; prelimbic/infralimbic) of male and female NAB and HAB rats on day 22 of treatment with either vehicle (Veh), minocycline (Min), escitalopram (Esc), or a combination of both (Min+Esc)**. Both HAB males (**a**) and females (**b**) showed overall reduced microglial numbers in the PFC compared to NAB rats independent of treatment. Min alone further reduced microglial numbers exclusively in male HAB rats (**a**). Representative microphotographs of the analyzed PFC with Iba+ microglia (green) and DAPI+ (blue) staining (**c**, **d**). Data represents mean + s.e.m., *p < 0.05 vs. corresponding Veh group; ++*p* < 0.01, +++*p* < 0.001 vs. NAB; two-way ANOVA, followed by a Bonferroni post hoc test; *n* = 6. Scale bar: 50 µm

**Fig. 3:**
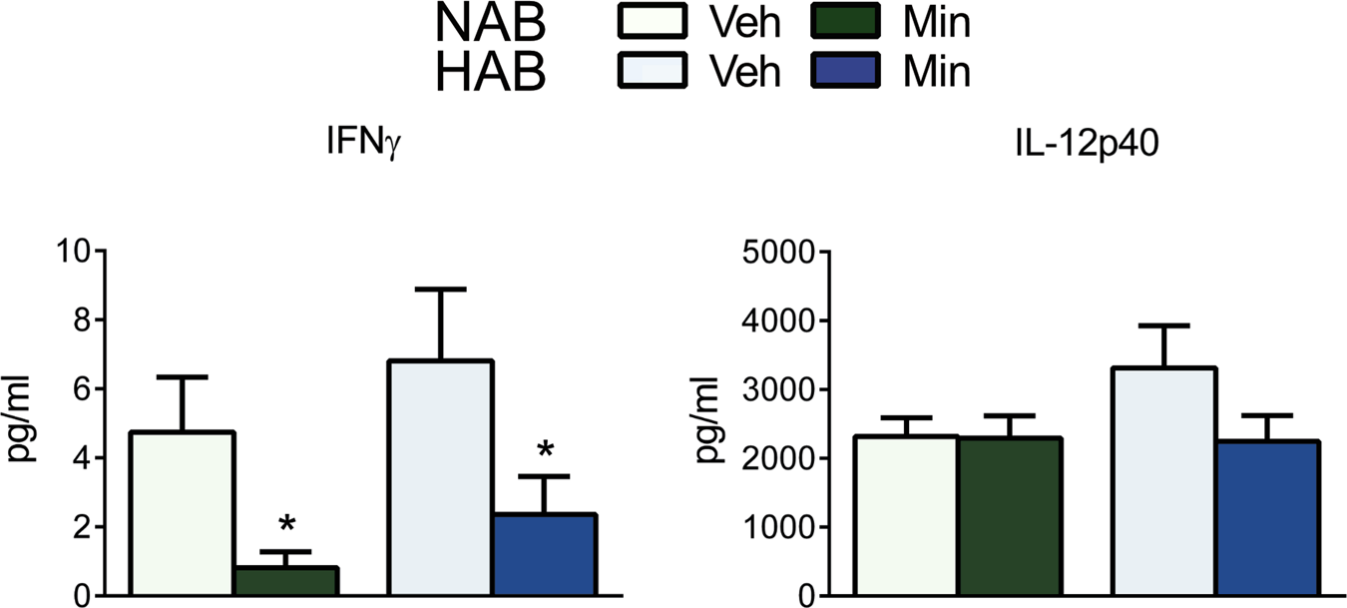
**Cytokine concentration in plasma samples of male HAB and NAB rats on day 22 of vehicle (Veh) or minocycline (Min) treatment**. Interferon (IFN)-γ and interleukin (IL)-12p40 levels were measured by a Luminex® bead immunoassay. Min reduced IFN-γ concentrations in both HAB and NAB rats (**a**), while IL-12p40 levels were not affected (**b**). Data represents mean + s.e.m., *p < 0.05 vs. corresponding Veh group; Mann–Whitney *U*-test

The effects of minocycline, escitalopram, or the combination on microglial quantity in infralimbic/prelimbic PFC slices were assessed using Iba-1 as a marker of both resting and reactive microglia. In male (*p* < 0.001; Fig. 2a) and female (*p* < 0.01; Fig. 2b) HAB rats, overall lower counts of microglia cells were detected compared to NAB rats. A treatment effect was seen exclusively in male HAB rats with post hoc analysis revealing further reduced microglial numbers after minocycline treatment (*p* < 0.05; Fig. 2a). As only male HAB rats responded both behaviorally and in microglial numbers to minocycline, all subsequent analyses, i.e. of peripheral pro-inflammatory cytokine concentrations and microbiome composition, were performed exclusively in minocycline-treated male HAB vs. NAB rats. Minocycline reduced IFN-γ levels in male HAB and NAB rats (*p* < 0.05; Fig. 3a), whereas IL-12 subunit p40 levels remained unchanged (Fig. 3b).

### Distribution of minocycline in liver and fecal boli

Minocycline was detectable in fecal boli suggesting its significant availability in the large intestine, including the cecum (Table 1). Comparable levels were found in the liver of both male and female rats irrespective of trait anxiety. This correlated with liquid (i.e. minocycline) intake on the last treatment day (data not shown).

**Table 1:** Minocycline concentration in liver and fecal boli of male and female rats

Sex	Treatment	Fecal boli [µg/mg]	Liver [µg/mg]
**♂**	VEH	0 ± 0	0 ± 0
	Minocycline	1.26 ± 0.11	1.05 ± 0.08
**♀**	VEH	n.a.	0 ± 0
	Minocycline	n.a.	1.12 ± 0.10

Data depicts mean concentration values ± s.e.m. of minocycline in tissue/stool of pooled HAB and NAB rats per sex; *n* (liver) = 13 (♀) or 36 (♂), *n* (fecal boli) = 6; n.a. = not assessed

### Intestinal microbial composition

**Fig. 4:**
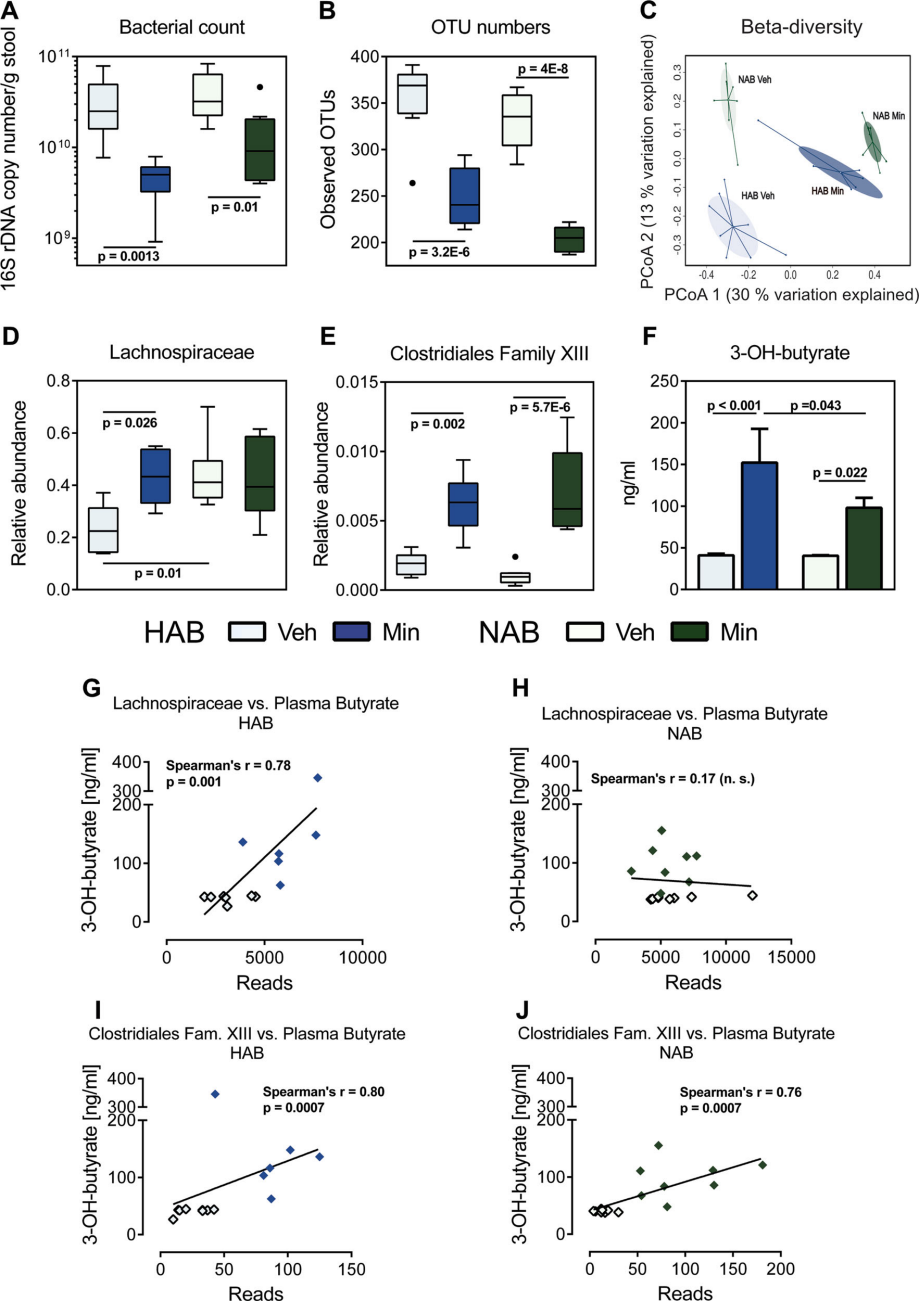
**Cecal microbiota composition and plasma 3-OH-butyrate levels after 22 days of vehicle (Veh) or minocycline (Min) treatment**. HAB and NAB rats showed comparable levels of 16S-rDNA copy numbers that were similarly diminished by Min (**a**) and comparable bacterial richness of the microbiome between both lines, as indicated by operational taxonomic units (OTU) numbers, that were decreased by Min (**b**). Beta-diversity of bacterial communities prevalent in cecal contents, as illustrated by principal coordinates analysis (PCoA) of Bray–Curtis distances with the first two coordinates plotted, showed four distinct clusters corresponding to all treatment groups (**c**). Connected symbols represent data from individual rats, color-coded by the indicated groups. Regarding Clostridiales families, HAB rats show a reduced abundance of Lachnospiraceae that was increased to NAB level by Min (**d**). Further, both lines show comparable levels of Clostridiales Family XIII with increased abundance after Min treatment (**e**). These changes were accompanied by increased plasma 3-OH-butyrate levels (**f**) with a higher increase in HAB compared to NAB rats. In HAB rats, plasma 3-OH-butyrate levels correlated positively with both Clostridiales families (**g**, **i**) whereas in NAB rats, a positive correlation was only observed with Family XIII (**h**, **j**; open symbols indicate Veh-treated, colored symbols Min-treated rats) as determined by Spearman’s correlation. Boxes in **a**, **b**, **d**, and **e** represent interquartile range with horizontal lines indicating the median of values, outliers indicate values more than 1.5 times of upper/lower quartile, and whiskers show minimum and maximum of remaining values. Bars represent mean + s.e.m; two-way ANOVA was used for statistical analysis

After 16S-rDNA-based high-throughput sequencing, analysis revealed comparable total bacterial 16S-rDNA copy numbers and microbial richness, represented by OTU counts, in HAB and NAB rats, which was reduced by minocycline in both lines (*p* < 0.01; Fig. 4a, b). The overall composition of cecal microbiota, as assessed by principal coordinates analysis of Bray–Curtis distances (beta-diversity), revealed marked differences between HAB and NAB rats shown by four distinct clusters corresponding to the two treatment conditions (*p* = 0.012; Fig. 4c). This analysis clearly shows a strong perturbation of the gut microbiota as a response to minocycline application in both rat lines and diminished differences between HABs and NABs following treatment.

In detail, striking differences between HAB and NAB rats were identified regarding the highly prevalent Lachnospiraceae family of the Clostridiales order (*p* < 0.05; Fig. 4d). Minocycline treatment elevated the lower frequency in HABs to the level of NABs that — in terms of this family — were not affected by treatment. Further, a relative increase in the abundance of the Clostridiales Family XIII in both lines was observed after minocycline (*p* < 0.01; Fig. 4e). Interestingly, many Lachnospiraceae members as well as a number of Clostridiales Family XIII strains have been implicated in the production of butyrate that exerts important pleiotropic functions in the intestinal tract and beyond^49^. Consistently, we found significantly augmented levels of 3-OH-butyrate after minocycline treatment in both lines that was even higher in HAB compared to NAB rats (*p* < 0.05; Fig. 4f). Spearman’s rank analysis disclosed positive correlations between 3-OH-butyrate levels and abundance of the two mentioned Clostridiales families in individual HAB rats corroborating an important contribution of the respective bacteria to systemic butyrate availability (*p* < 0.001; Fig. 4g, i). In NAB rats, minocycline-induced augmentation of the Family XIII population, but not of Lachnospiraceae, positively correlated with 3-OH-butyrate levels (*p* < 0.001; Fig. 4h, j), which presumably accounted for the moderate increase in 3-OH-butyrate concentrations of NABs.

## Discussion

In the present study, we confirm the antidepressant effect of minocycline and reveal that chronic minocycline treatment reduces depressive-, but not anxiety-like, behavior and affects the gut–brain axis in a sex- and trait anxiety-dependent manner. These findings extend previous reports of antidepressant actions of minocycline^18,20,25,26,28,50^ to male rats with high levels of innate anxiety- and depressive-like behavior. In addition, chronic minocycline facilitated social preference behavior in HAB males and females as indicated in previous studies^25,51,52^. However, as HAB rats generally do not show impairments in social interaction, the elevated social preference rather seems to reflect an alleviation of depressive-like behavior^36,53^ than an improvement in social approach per se. Interestingly, we could not replicate the proposed anxiolytic-like effect of minocycline^17,50^ either in HAB or NAB rats. Thus, it appears that anxiety-like behavior in HAB rats is manifested via a mechanism independent of the inflammatory system or any other target of minocycline^33,54^. Importantly, the antidepressant effect of minocycline was not observed in female HAB rats. Similarly, minocycline was able to abolish inflammatory neuropathic pain^55^ and improved morphine-induced analgesia^56^ only in male mice, proposing a sex-specificity of its actions. Of note, we carefully considered an influence of repeated testing^57,58^ on the behavioral effects of minocycline and, accordingly, the least stressful test (SPT) was performed first, and the most stressful test (FST) last, with a full day of rest between two tests to avoid stress- or experience-induced bias.

Chronic escitalopram^59^ or citalopram^60^ treatment has been described to ameliorate chronic stress-induced depressive-like behavior in both rats and mice, and citalopram and paroxetine given over 8 weeks have also been proven to be effective in HAB males^34,35^. In the present study we could only reproduce the anxiolytic effect of escitalopram in male NAB rats in the LDB, but not in HAB or female rats, probably due to the short treatment time. Importantly, these results support a sex difference in treatment strategies for MDD and validate the HAB breeding line as a model for innate treatment-resistant depressive-like behavior with minocycline as a potential treatment.

Clinical studies provide growing evidence for an increased efficacy of adjunctive treatment in MDD^5^. Fittingly, minocycline treatment, in combination with several conventional antidepressants like SSRIs or antipsychotics, improved MDD symptoms^19–21^. However, a study in depressed patients revealed that administration of citalopram in combination with a non-steroidal anti-inflammatory drug inhibited remission^61^. In keeping, in the present study 3-week treatment with minocycline together with escitalopram abolished the antidepressant effect of minocycline. In agreement with our results, minocycline in combination with the SSRI fluoxetine, but not the tricyclic desipramine, prevented the antidepressant effects of minocycline in rats^23,24^. Together, these findings strongly suggest that minocycline specifically counteracts a mechanism responsible for the antidepressant-like effect of SSRIs. Though minocycline interferes with the effectiveness of escitalopram in this case, it does not necessarily target the monoaminergic mode of action, but might target substance-specific secondary mechanisms or elicit local pharmacokinetic interactions.

Minocycline has been repeatedly demonstrated to inhibit microglial activity and proliferation accompanying its effects on behavior^16,17,26,28,43,50,62,63^ and - as an antibiotic - to modulate the gut microbial composition^18^. Neuroinflammation and increased microglial activation have been associated with MDD^8,27,43,64,65^. However, a dynamic and bi-directional alteration in microglial status has recently been hypothesized to underlie depressive-like behavior, as microglial numbers were found to be initially elevated followed by their decline in the hippocampus and PFC of chronically stressed rodents that displayed depressive-like behavior^51^. The lower microglial density in the PFC of HAB rats is likely due to their permanent stress state, as shown by elevated trait anxiety and a hyper-reactive HPA axis^66^. Interestingly, administration of minocycline further reduced microglial numbers, specifically in male HAB rats, which paralleled the antidepressant-like behavioral effect. Similar to a reduction in microglia, acute inhibition of the infralimbic cortex in male HAB rats led to an antidepressant-like phenotype^39^, indicating the PFC to be involved in depressive-like behavior in male HAB rats. Minocycline has been shown to inhibit microglial proliferation^17,67^ and, in view of a constant rate of microglial apoptosis^68^, should therefore lead to a further reduced microglial density in male HAB rats. Although the underlying mechanisms of the dynamic interplay between proliferation and apoptosis need to be explored in more detail, our results point towards an impaired microglial homeostasis and functioning as an underlying mechanism for the depressive-like behavior in HAB rats, and further support our breeding line as a model for inflammation-associated depressive-like behavior. Of note, the reduction in microglial numbers by minocycline was abolished, when given in combination with escitalopram.

The microbiome-gut–brain axis has been shown to modulate microglia proliferation and maturation. For example, phenotypical abnormalities of microglia found in germ-free mice were reversed by a microbiota-derived short-chain fatty acid mixture containing butyrate^12^. Gut microbiota dysbiosis, in turn, can result in neuroinflammation by chronic activation of microglia in the CNS^69^, potentially contributing to the behavioral phenotype in HAB rats. Supporting the link between the microbiome and emotional behavior^6,9,70^, HAB and NAB rats originating from the same breeding facility show - despite comparable total bacterial numbers in their cecal contents - profound differences in global gut microbiota composition, reflecting the robust behavioral differences between the two lines. As expected, 3 weeks of minocycline treatment reduced bacterial richness similarly in both groups resulting in still persisting, but diminished group differences. This suggests a partial equalization of the microbiota between both lines that might contribute to the observed antidepressant-like effect of minocycline in HAB rats. Interestingly, in patients suffering from MDD, an altered microbial composition was observed that induced similar symptoms in rats upon transplantation^71,72^.

Importantly, detailed microbiome comparison between HAB and NAB rats revealed decreased relative abundance of the Lachnospiraceae family in HABs, which expanded after minocycline treatment. Similar dynamic changes in Lachnospiraceae were found in a stress model of depression^18^. We further show that minocycline elevated relative Clostridiales Family XIII levels in both HAB and NAB rats. Both Lachnospiraceae and Clostridiales Family XIII accommodate a large part of butyrate-producing bacterial genera, reflected by increased plasma 3-OH-butyrate levels especially in HAB, but also in NAB rats, after minocycline. In support, a positive correlation between the abundance of Lachnospiraceae, as well as the Clostridiales Family XIII, with 3-OH-butyrate was found in male HAB rats. In contrast, in male NABs a positive correlation was only found with Clostridiales Family XIII. Both butyrate and 3-OH-butyrate have been proposed as anti-inflammatory agents that execute their effects via inhibition of peripheral T cell activity^73^ and microglia activation^74^, and even via inducing microglial apoptosis^75^. In line, minocycline decreased the level of the pro-inflammatory cytokine IFN-γ, produced by IL-12-induced and activated T-helper type 1 cells^76^, in both HAB and NAB males. Plasma levels of IL-12p40 appeared to be higher in HAB rats and were reduced by minocycline to NAB levels, supporting a regulatory role of minocycline and butyrate on peripheral T cells^73,77^ independent of the central immune system that might contribute to the antidepressant effect of minocycline. Although a role of butyrate in the regulation of the central immune system remains to be studied, our results suggest that butyrate-mediated microglial apoptosis^75^ might contribute to the observed reduction in microglial density in the PFC of male HAB rats. Furthermore, butyrate has been shown to exert antidepressant-like effects in mice^78,79^ and rats^80^. These findings point towards an important role of bacteria-produced butyrate as a mediator of behavioral and microglial alterations in minocycline-treated HAB rats. Thus, our results support the hypothesis of an influence of the gut microbiome on anxiety- and depressive-like behavior in our rodent model of anxiety and depression.

In summary, we show that the depressive- and anxiety-like phenotype of HAB rats is accompanied by reduced microglial numbers in the infralimbic/prelimbic PFC and a distinct shift of the gut-bacterial composition. This emphasizes that HAB rats are a valid model for inflammation-associated depressive-like behavior and points towards a complex interplay between microbiota/microbial metabolites and the immune system, as proposed by the concept of the microbiota-gut–brain axis.

In the course of clinical application, the present results indicate that promoting an anti-inflammatory state by control of microglial activation and/or modulation of the gut microbiome and metabolome, as shown after prolonged minocycline treatment, offers promising potential as a therapeutic strategy in treatment-resistant MDD. The present study was designed to detect an early response (3 weeks) to minocycline and did not mimic a clinical antidepressant treatment. Thus, a longer treatment duration might reveal different effects. Nevertheless, caution is needed when considering minocycline as an augmentation strategy of SSRIs like escitalopram, especially in the absence of a pro-inflammatory profile, which may explain some of the recent high-profile clinical failures of minocycline.

## Supplementary information

Supplementary methods and results
