# Supplementary material for: Minocycline alters behavior, microglia and the gut microbiome in a trait-anxiety-dependent manner
Source: Transl Psychiatry. 2019 Sep 13;9:223. doi: 10.1038/s41398-019-0556-9 (PMC6744405; doi:10.1038/s41398-019-0556-9)
Supplement: Supplementary file 1 — Supplementary methods and results [file 41398_2019_556_MOESM1_ESM.docx]

**Supplementary information**

**Supplementary Methods**

**Behavioral experiments**

Behavioral experiments were performed three times in independent sets of animals.

Social preference test (SPT): To test for social motivation and social interactions, the SPT was performed in a novel arena as previously described ^1^. Rats were placed in an arena (40 x 80 x 40 cm), and after 30 s of habituation, an empty wire-mesh cage (non-social stimulus; 20 x 9 x 9 cm) was placed on one short side wall for 4 min for free exploration. The empty cage was then replaced by an identical one containing an unknown sex- and weight-matched con-specific (social stimulus) for an additional 4 min period. Before each trial, the arena was cleaned thoroughly with water containing a low concentration of detergent. Each trial was videotaped, and the investigation times of the non-social and social stimuli were scored using JWatcher behavioral observation software (V 1.0, Macquarie University and UCLA).

Light-dark box (LDB): To test for anxiety-related behavior and locomotion, the LDB was performed as previously described ^2^. Briefly, the LDB consists of a light (40 x 50 cm, 100 lux; light box) and a dark (40 x 30 cm, 0 lux) compartment connected *via* a small opening (7.5 x 7.5 cm) to enable transition between the boxes. Rats were placed into the light box facing away from the opening and the 5 min test was recorded *via* an overhead camera. Time spent in the light compartment as indicator for anxiety-like behavior and total distance traveled were assessed using NOLDUS software (Ethovision XT Version 12, Netherlands).

Elevated plus-maze (EPM): As an additional test for anxiety-related behavior, rats were tested on the EPM ^3^. The EPM consisted of two closed arms (50 x 10 x 40 cm; 10 lux) and two open arms (50 x 10 cm; 40 lux) connected by a central neutral zone (10 x 10 cm) that are elevated 70 cm from the floor. Rats were placed into the neutral zone facing one closed arm and the 5 min test was recorded by an overhead camera. The percentage time spent on the open arms was analyzed as an indicator for anxiety.

Forced swim test (FST): To assess active versus passive stress coping with the latter being an indicator of depression-like behavior, rats were tested in the FST as previously described ^4–6^. Rats were individually placed into a plexiglass cylinder (21 x 46 cm) filled with water (23-25 °C) to a depth of 30 cm for 15 min (pre-swim), dried and returned to their home cage. After 24 h, rats were replaced into the swim cylinder under the same conditions for 5 min (FST). Water was changed between each rat, and both sessions were recorded using an overhead camera placed above the cylinder. The predominant behavior (struggling, swimming or immobility) during each 5-s period of the FST was rated using a time sampling technique, providing a total of 60 scores per rat.

**Perfusion and tissue preparation**

Transcardial perfusion was performed with ice-cold 0.01 M phosphate buffered saline (1 x PBS; pH 7.4) followed by 4 % paraformaldehyde (PFA; pH 7.4). During the PBS perfusion, blood, microbiome samples from the caecum and fecal boli, and liver samples were collected. Blood was centrifuged for 10 min at 5000 x g and plasma, liver, fecal boli, as well as cecal samples were frozen using dry ice and stored at -80 °C until further analysis. Brains were removed, postfixed for 24 h in 4 % PFA at 4 °C and cryo-protected in 30 % sucrose at 4 °C. After 3 days, brains were snap-frozen, coronal cryo-sections (40 µm) were cut and stored in 30 % ethylene glycol/30 % glycerol (Sigma-Aldrich, Germany) in 1 x PBS at -20 °C until further processing.

**Immunofluorescent-immunohistochemistry**

Immunofluorescent-immunohistochemistry was performed in two independent sets of animals.

For Immunofluorescent-immunohistochemistry identification of microglia using ionized calcium-binding adapter molecule-1 (Iba-1), brain sections from the infralimbic and prelimbic PFC were washed thoroughly in 1 x PBS, before permeabilization and blocking for 1 h in 0.1 % Triton-X 100 and 2 % Normal Goat Serum (NGS; Vector Labs, Biozol) in 1 x PBS at room temperature. Afterwards, slices were incubated with the primary rabbit anti-Iba-1 antibody (1:1000, WAKO, 019-19741) in blocking solution over night at 4°C, washed and incubated with the corresponding secondary antibody anti-rabbit Alexa Fluor 488 (1:1000, Invitrogen) together with DAPI (1:1000, Sigma-Aldrich) in 2 % NGS in 1 x PBS for additional 2 h. Finally, all sections were washed and mounted on slides with Aquapolymount for confocal analysis.

Quantification of microglia cells was performed in 6 brains randomly selected of each treatment condition using confocal microscopy (Olympus confocal microscope inverted type IX81, Olympus Europe Holding GmbH, Hamburg, Germany). From an average of 5 images (20 optical sections, 1 µm Z-step size), Iba-1-positive cells (Iba+) and total cells (DAPI+) were counted using the ImageJ plugin “cell counter” and the mean percentage of microglia was calculated by normalization to DAPI+ cells to account for potential differences in cell densities due to perfusion-dependent tissue shrinkages.

**Minocycline tissue extraction and HPLC analyses**

A sensitive and specific reversed-phase high-performance liquid chromatographic (HPLC) method was developed and optimized for rapid resolution and sensitive detection of minocycline isolated from rat tissues. Minocycline concentrations were assessed in liver tissue and fecal boli. Samples were homogenized in calcium-free PBS at a ratio of 1:5 (w/v) by 30 strokes in glass-PTFE potter homogenizers. Homogenates were subsequently acidified by adding 0.02 vol of orthophosphoric acid (85%, w/v), centrifuged (1000*g, 5 min, 4 °C) and supernatant was loaded onto HLB Oasis column (30 mg; Waters, Milford MA, USA). Columns were washed three times with 1 ml of 5% methanol in ultrapure water, eluted at atmospheric pressure in 1 ml methanol and evaporated to dryness in a heat block set to 50 °C under shielding gas (Argon). Residues were reconstituted in HPLC mobile phase, centrifuged (5 min. 15.000 * g) and injected directly onto a C8 Inertsil column (GL Sciences, Tokyo, Japan). HPLC analysis was performed using a Shimadzu 10-series system with isocratic elution at 1.4 ml/min and UV detection at 350 nm. The mobile phase contained acetonitrile-methanol-ultrapure water-acetic acid (2.5:10:85:2.5, v/v). The detection limit was 50 ng/ml. Recovery from liver homogenates and fecal boli averaged 95%, intra- and inter-assay coefficient of variation averaged about 5% and 10% at 5 µg/ml. All chemicals were purchased from Sigma-Aldrich.

#### Luminex^®^ cytokine detection assay

To quantify interferon (IFN)-γ and interleukin (IL)-12p40, a rat cytokine bead immunoassay (Invitrogen/ThermoFisher Scientific, Darmstadt, Germany) was used. All reagents required were provided with the kit and were prepared according to the manufacturer’s protocol. Plasma samples were pre-diluted 3-fold in assay diluent and applied in a volume of 50 µl. Acquisition of raw data was performed by the *Luminex xMAP 100* system (Luminex, Austin, TX, USA). The software was set to acquire data using 75 µl of sample per well and to count 100 events per single bead set. Raw data was captured as mean fluorescence intensity, and the concentration of IFN-γ and IL-12p40 in the samples was calculated based on 4- or 5-parameter logistic fit standard curves using the *LiquiChip* Analyzer software (Qiagen, Hilden, Germany).The lower detection limit was 2 pg/ml (IFN-γ) and 10 pg/ml (IL-12p40), respectively.

**Intestinal microbiome analysis by 16S-rDNA pyrosequencing**

Isolation of DNA from stool specimens*:* Cecum contents was collected from male HAB and NAB rats, immediately cooled on dry ice and stored at -80 °C until processing. After thawing, samples (100 mg wet weight each) were mixed with a pool of three spike bacteria (*Salinibacter ruber, Rhizobium radiobacter, Alicyclobacillus acidiphilus*) containing a defined number of 16S-rDNA copies. Lysis of cells was performed by exposure to S.T.A.R. Buffer (Roche, Mannheim, Germany)/proteinase K, five cycles of freezing in liquid nitrogen and boiling, and repeated bead beating in the TissueLyser II (Qiagen). DNA was purified by means of the MagNA Pure 96 instrument (Roche) using the MagNA Pure 96 DNA and Viral NA Large Volume Kit (Roche). Quantification of total nucleic acids and dsDNA was carried out by using the NanoDrop 1000 spectrophotometer (Thermo Fisher Scientific, Wilmington, DE, USA) and the dsDNA-specific Quant-iT PicoGreen reagent (Invitrogen/Thermo) in a VICTOR^3^ fluorescence reader (PerkinElmer, Waltham, MA, USA), respectively.

Quantification of 16S-rDNA copies by qPCR: 16S-rRNA gene copy numbers of total bacteria in the isolated DNA were determined by qPCR on a LightCycler 480 II Instrument (Roche). PCR reactions included universal eubacterial 16S-rRNA gene primers 764F and 907R and the LightCycler 480 SYBR Green I Master kit (Roche). Additionally, spike bacteria-specific qPCRs were performed to identify errors in DNA isolation before undergoing amplification and pyrosequencing (data not shown, primers and probes are specified in Supplementary table S1).

**Supplementary table S1. Primers used for quantification of 16S-rDNA copy numbers by qPCR and for pyrosequencing.**

| Name | DNA sequence (5’ - 3’) | *Escherichia coli* 16S-rDNA nucleotide position | Purpose of use | Ref |
| --- | --- | --- | --- | --- |
| 764F | caaacaggattagataccc | 764 | Quantification of total 16S-rDNA copies | ^7^ |
| 907R | ccgtcaattcctttragttt | 907 | Quantification of total 16S-rDNA copies | ^8^ |
| 341F | CCATCTCATCCCTGCGTGTCTCCGACTCAG<MID>cctacgggaggcagcag | 341 | Pyrosequencing | ^9^ |
| 1061R | CCTATCCCCTGTGTGCCTTGGCAGTCTCAGcrrcacgagctgacgac | 1061 | Pyrosequencing | ^9^ |
| Aacidi-238TM | 6FAM-agctagttggtgaggtaacggcccacc-BBQ | 238 | Quantification of 16S-rDNA copies of *Alicyclobacillus acidiphilus* | ^10^ |
| Aacidi-193F | gaggaaagttgcaaatgcaaca | 193 | Quantification of 16S-rDNA copies of *Alicyclobacillus acidiphilus* | ^10^ |
| Aacidi-453R | aggagctttccactctccttat | 453 | Quantification of 16S-rDNA copies of *Alicyclobacillus. acidiphilus* | ^10^ |
| Rradio-166TM | LC670-aattaataccgcatacgccctacg-BBQ | 166 | Quantification of 16S-rDNA copies of *Rhizobium radiobacter* | ^10^ |
| Rradio-126-F2 | ggaacataccctttcctgcgg | 126 | Quantification of 16S-rDNA copies of *Rhizobium radiobacter* | ^10^ |
| Rradio-197-R2 | gccaatccttccccgataaatc | 197 | Quantification of 16S-rDNA copies of *Rhizobium radiobacter* | ^10^ |
| Salini-180TM | LC640-cacgtcgtctggatcccgcatg-BBQ | 180 | Quantification of 16S-rDNA copies of *Salinibacter ruber* | ^10^ |
| Salini-7F | agagtttgatcatggctcag | 7 | Quantification of 16S-rDNA copies of *Salinibacter ruber* | ^10^ |
| Salini-413R | tacgccccataggggtgt | 413 | Quantification of 16S-rDNA copies of *Salinibacter ruber* | ^11^ |

Amplification of V3-V6 16S-rDNA variable region and 454 pyrosequencing: V3 to V6 hypervariable regions of bacterial 16S-rRNA genes were amplified from a total of 10 ng dsDNA using the forward primer 341F containing a 10-bp multiplex identifier (MID) sequence, and the reverse primer 1061R. The resulting 790-bp amplicons were recovered from gels by use of the QIAquick Gel Extraction kit (Qiagen, Hilden, Germany) and further purified with Agencourt AMPure XP beads (Beckman Coulter, Krefeld, Germany). Copy numbers of amplicons containing Lib-L-adaptors were determined using the KAPA Library Quant 454 Titanium/Lib-L Universal Kit (KAPA Biosystems, Wilmington, DE, USA). To prepare the DNA library, equimolar concentrations of 1 x 10^6^ adaptor-labeled amplicon molecules/µl for each sample were pooled. This library was re-amplified by emulsion PCR using the GS FLX Titanium LV emPCR kit (Lib-L) and thereafter subjected to sequencing on a GS FLX+ instrument (454/Roche) with the GS FLX Titanium Sequencing Kit XL+ using acyclic flow pattern B.

Sequence processing and operational taxonomic unit (OTU) clustering: A total of 1,398,617 (917 Mb) processed sequencing reads, which passed quality filters with a median read length of 677 bases, were further processed using a combination of QIIME ^12^ (v1.9.1) and R ^13^ (version 3.4.0) with installed Bioconductor package ^14^. Reads were de-multiplexed and filtered for quality using QIIME’s *split_libraries.py* script with default parameters except minimum and maximum read length, which were set to 400 bp and 800 bp, respectively. The filtered reads were mapped to OTUs built on the SILVA database (release 128) ^15^ using QIIME’s implementation of the UCLUST 1.2.21 ^16^ algorithm at a 97% sequence identity threshold. Raw sequencing data has been deposited in the European Nucleotide Archive (ENA) (http://www.ebi.ac.uk/ena/ data/view/PRJEB30124).

Microbial composition and global community structure analysis: Classified reads were grouped by taxonomy to generate box plots showing relative abundances on various levels. The richness, represented by the number of observed OTUs, as well as the diversity between samples, characterized by a Bray-Curtis dissimilarity matrix, were calculated and plotted with the *vegan* *2.4-1* package of *R*.

**Supplementary Results**

**Supplementary table S2. Statistical analysis of behavioral, plasma cytokine, 3-OH-butryate, and microglia data and detailed n numbers for each experimental group.** All statistical tests were followed by a Bonferroni post hoc correction for multiple comparisons if appropriate. N.a. = not assessed

|  | Effects of minocycline on behavior  One-way ANOVA for repeated measures (SPT) or two-way ANOVA (FST) | | | |
| --- | --- | --- | --- | --- |
| sex | SPT | Stimulus effect | Stimulus x treatment effect | |
| ♂ | HAB (Fig. 1A) | F_(1,41)_ = 169.961; p<0.001 * | F_(3,41)_ = 2.483; p = 0.07 (*) | |
| ♀ | HAB (Fig. 1B) | F_(1,42)_ = 233.512; p<0.001 * | F_(3,42)_ = 2.530; p = 0.07 (*) | |
| ♂ | NAB (Fig. 1C) | F_(1,40)_ = 78.364; p<0.001 * | F_(3,40)_ = 0.36; p = 0.78 | |
| ♀ | NAB (Fig. 1D) | F_(1,28)_ = 208.968; p<0.001 * | F_(3,28)_ = 0.805; p = 0.50 | |
|  | FST | Line effect | Line x treatment effect | |
| ♂ | Fig. 1E | F_(1,84)_ = 27.618; p<0.001 * | F_(3,84)_ = 4.082; p = 0.009 * | |
| ♀ | Fig. 1F | F_(1,67)_ = 21.168; p<0.001 * | F_(3,67)_ = 0.024; p = 0.99 | |
| ♂ | Fig. 1G | F_(1,84)_ = 16.496; p<0.001 * | F_(3,84)_ = 3.316; p = 0.024 * | |
| ♀ | Fig. 1H | F_(1,67)_ = 22.978; p<0.001 * | F_(3,67)_ = 0.237; p = 0.87 | |
|  | **Effects of minocycline on microglia cells**  **Two-way ANOVA** | | | |
|  |  | Line effect | Line x treatment effect | |
| ♂ | Fig. 2A | F_(1,40)_ = 70.59; p<0.001 * | F_(3,40)_ = 2.894; p<0.05 * | |
| ♀ | Fig. 2B | F_(1,40)_ = 7.616; p<0.01 * | F_(3,40)_ = 0.272; p = 0.845 | |
|  | **Effects of minocycline on plasma cytokine concentrations (n = 8 – 14 per group)**  **Mann-Whitney-U test** | | | |
|  |  | Treatment effect | | |
|  |  | HAB | | NAB |
| ♂ | Fig. 3A | U = 48.000; p = 0.012 * | | U = 71.000; p = 0.032 * |
| ♂ | Fig. 3B | U = 89.000; p = 0.505 | | U = 115.000; p = 0.642 |
|  | **Effects of minocycline on plasma 3-OH-butyrate (n = 6 – 8 per group)**  **Two-way ANOVA** | | | |
|  |  | Line effect | | Line x treatment effect |
| ♂ | Fig. 4F | F_(1,26)_ = 4.486; p < 0.001 * | | F_(1,26)_ = 2.396; p = 0.134 |
|  | **Effects of minocycline on anxiety-like behavior (n = 8 – 14 per group)**  **Two-way ANOVA (LDB & EPM)** | | | |
|  | LDB | Line effect | Line x treatment effect | |
| ♂ | Fig. S2A | F_(1,84)_ = 35.729; p<0.001 * | F_(3,84)_ = 2.030; p = 0.12 | |
| ♀ | Fig. S2B | F_(1,68)_ = 113.024; p<0.001 * | F_(3,68)_ = 1.785; p = 0.16 | |
| ♂ | Fig. S2C | F_(1,81)_ = 47.115; p<0.001 * | F_(3,81)_ = 0.379; p = 0.769 | |
| ♀ | Fig. S2D | F_(1,68)_ = 39.970; p<0.001 * | F_3,68)_ = 0.557; p = 0.645 | |
|  | EPM | Line effect | Line x treatment effect | |
| ♂ | Fig. S2C | F_(1,81)_ = 54.702; p<0.001 * | F_(3,81)_ = 1.080; p = 0.36 | |
| ♀ | Fig. S2D | F_(1,70)_ = 180.528; p<0.001 * | F_(3,70)_ = 0.875; p = 0.46 | |
|  | **Effects of a higher dose of minocycline on behavior (n = 8 – 10 rats per group)**  **One-way ANOVA for repeated measures (SPT) or student’s t-test (LDB, EPM & FST)** | | | |
|  | SPT | Stimulus effect | Stimulus x treatment effect | |
| ♂ | Fig. S3A | F_(1,16)_ = 190.714; p<0.001 * | F_(1,16)_ = 14.798; p = 0.001 * | |
| ♀ | Fig. S3B | F_(1,15)_ = 97.423; p<0.001 * | F_(1,15)_ = 32.958; p<0.001 * | |
|  | LDB | Treatment effect | | |
| ♂ | Fig. S3C | T_(16)_ = 0.912; p = 0.38 | | |
| ♀ | Fig. S3D | T_(15)_ = 1.160; p = 0.26 | | |
|  | EPM |  |  | |
| ♂ | Fig. S3E | T_(17)_ = 1.581; p = 0.13 | | |
| ♀ | Fig. S3F | T_(16)_ = -0.660; p = 0.52 | | |
|  | FST |  |  | |
| ♂ | Fig. S3G | T_(18)_ = -2.427; p<0.05 * | | |
| ♀ | Fig. S3H | T_(14)_ = 0.554; p = 0.59 | | |
|  |  |  | | |

| **Group sizes** | | | | | | | |
| --- | --- | --- | --- | --- | --- | --- | --- |
| trait | sex | Treatment | behavior | Microglia | cytokines | 3-OH-butyrate | microbiome |
| 40 mg / kg / day minocycline | | | | | | | |
| HAB | ♂ | Veh | 14 | 6 | 14 | 8 | 8 |
|  |  | Min | 15 | 6 | 15 | 6 | 6 |
|  |  | Esc | 9 | 6 | n.a. | n.a. | n.a. |
|  |  | Min+Esc | 10 | 6 | n.a. | n.a. | n.a. |
|  | ♀ | Veh | 13 | 6 | n.a. | n.a. | n.a. |
|  |  | Min | 14 | 6 | n.a. | n.a. | n.a. |
|  |  | Esc | 10 | 6 | n.a. | n.a. | n.a. |
|  |  | Min+Esc | 11 | 6 | n.a. | n.a. | n.a. |
| NAB | ♂ | Veh | 16 | 6 | 16 | 8 | 8 |
|  |  | Min | 16 | 6 | 16 | 8 | 8 |
|  |  | Esc | 8 | 6 | n.a. | n.a. | n.a. |
|  |  | Min+Esc | 8 | 6 | n.a. | n.a. | n.a. |
|  | ♀ | Veh | 8 | 6 | n.a. | n.a. | n.a. |
|  |  | Min | 8 | 6 | n.a. | n.a. | n.a. |
|  |  | Esc | 8 | 6 | n.a. | n.a. | n.a. |
|  |  | Min+Esc | 8 | 6 | n.a. | n.a. | n.a. |
| 80 mg / kg / day minocycline | | | |  |  |  |  |
| HAB | ♂ | Veh | 10 | n.a. | n.a. | n.a. | n.a. |
|  |  | Min | 10 | n.a. | n.a. | n.a. | n.a. |
|  | ♀ | Veh | 9 | n.a. | n.a. | n.a. | n.a. |
|  |  | Min | 8 | n.a. | n.a. | n.a. | n.a. |

**Supplementary table S3. Fluid intake on average per animal [ml].** Data is presented as mean (s.e.m.).

|  |  |  | Treatment day | | | | | | | | | | |
| --- | --- | --- | --- | --- | --- | --- | --- | --- | --- | --- | --- | --- | --- |
| group | Treatment | n | 1 | 2 | 14 | 15 | 16 | 17 | 18 | 19 | 20 | 21 | 22 |
|  |  |  |  |  |  | **SPT** |  | **LDB** |  | **EPM** |  | **Pre-swim** | **FST** |
| HAB ♂ | Veh | 5 | 29.7 (3.5) | 29.6 (2.3) | 30.5 (2.6) | 31.1 (2.2) | 28.3 (2.9) | 31.5 (1.9) | 28.3 (1.6) | 30.7 (2.5) | 29.3 (2.4) | 30.3 (1.6) | 29.0 (2.0) |
|  | Min | 5 | 28.5 (1.0) | 26.5 (2.1) | 31.0 (2.9) | 31.4 (2.7) | 30.5 (1.4) | 27.9 (1.1) | 25.4 (2.4) | 29.3 (2.1) | 30.1 (1.4) | 27.0 (2.7) | 29.3 (1.6) |
|  | Esc | 3 | 29.3 (2.1) | 22.7 (4.0) | 27.1 (3.5) | 28.9 (2.4) | 29.0 (1.0) | 30.9 (1.3) | 26.9 (0.9) | 31.4 (1.4) | 29.3 (2.5) | 28.7 (1.3) | 27.2 (2.3) |
|  | Min+Esc | 3 | 29.4 (2.8) | 24.0 (1.7) | 27.2 (3.0) | 28.1 (3.2) | 24.9 (0.4) | 27.7 (2.5) | 25.4 (1.2) | 28.4 (0.9) | 23.8 (1.4) | 24.8 (2.0) | 25.9 (2.1) |
| HAB ♀ | Veh | 4 | 21.2 (2.0) | 21.4 (2.1) | 22.6 (1.6) | 22.0 (3.0) | 22.2 (1.8) | 24.3 (3.1) | 22.5 (2.1) | 22.5 (2.7) | 22.4 (2.2) | 21.6 (1.8) | 23.5 (3.0) |
|  | Min | 4 | 21.9 (1.5) | 22.3 (1.5) | 24.8 (1.7) | 24.0 (0.5) | 23.6 (1.2) | 23.4 (0.7) | 21.3 (2.2) | 25.0 (0.5) | 21.8 (0.6) | 23.3 (0.7) | 21.5 (1.7) |
|  | Esc | 3 | 23.3 (0.8) | 21.4 (3.1) | 16.3 (1.2) | 20.0 (0.5) | 18.1 (2.3) | 21.3 (2.7) | 20.5 (2.9) | 22.7 (1.0) | 17.8 (1.5) | 17.6 (3.5) | 21.8 (0.8) |
|  | Min+Esc | 3 | 25.4 (0.6) | 25.1 (1.4) | 23.7 (0.6) | 23.0 (2.0) | 21.8 (1.5) | 23.7 (1.0) | 20.6 (2.1) | 23.8 (2.9) | 23.5 (3.7) | 23.6 (1.6) | 19.9 (0.9) |
| NAB ♂ | Veh | 4 | 44.1 (2.7) | 40.5 (2.4) | 43.9 (3.4) | 42.8 (1.5) | 44.4 (2.5) | 48.9 (1.3) | 41.7 (4.2) | 49.1 (2.4) | 42.8 (3.2) | 44.9 (3.1) | 43.5 (4.1) |
|  | Min | 4 | 45.3 (3.9) | 42.3 (3.8) | 51.4 (5.1) | 50.6 (3.7) | 46.0 (5.3) | 49.1 (4.1) | 43.1 (3.1) | 45.5 (2.8) | 46.8 (4.8) | 47.9 (3.2) | 49.7 (4.0) |
|  | Esc | 2 | 38.5 (1.8) | 39.8 (2.0) | 27.1 (18.9) | 40.3 (1.5) | 41.8 (3.5) | 43.4 (2.1) | 43.8 (0.3) | 43.0 (3.5) | 46.8 (2.8) | 44.3 (1.0) | 48.1 (2.9) |
|  | Min+Esc | 2 | 38.8 (3.5) | 39.9 (1.9) | 45.3 (1.3) | 39.9 (5.1) | 42.5 (5.0) | 44.0 (2.3) | 38.1 (1.6) | 42.4 (3.9) | 37.5 (1.0) | 41.3 (2.5) | 41.5 (2.0) |
| NAB ♀ | Veh | 2 | 42.3 (8.5) | 45.1 (14.4) | 36.0 (4.8) | 41.0 (8.3) | 41.8 (7.5) | 43.6 (8.1) | 36.6 (5.9) | 41.3 (3.3) | 41.1 (11.4) | 42.8 (10.3) | 45.5 (n.a.) |
|  | Min | 2 | 33.5 (4.5) | 32.3 (2.3) | 32.3 (5.8) | 36.1 (2.6) | 37.0 (0.5) | 36.9 (1.4) | 33.8 (1.8) | 30.0 (0.8) | 36.8 (1.3) | 31.3 (1.5) | 32.3 (0.3) |
|  | Esc | 2 | 41.0 (2.5) | 29.1 (1.4) | 26.1 (4.1) | 25.6 (4.4) | 22.4 (0.4) | 26.3 (1.3) | 25.8 (3.0) | 23.8 (4.8) | 24.6 (0.6) | 22.1 (2.9) | 23.8 (3.8) |
|  | Min+Esc | 2 | 36.1 (1.1) | 33.8 (1.3) | 28.3 (0.8) | 31.1 (1.4) | 33.1 (1.9) | 34.4 (0.1) | 30.9 (1.1) | 31.0 (1.0) | 32.5 (0.3) | 29.0 (0.5) | 31.3 (3.0) |

**
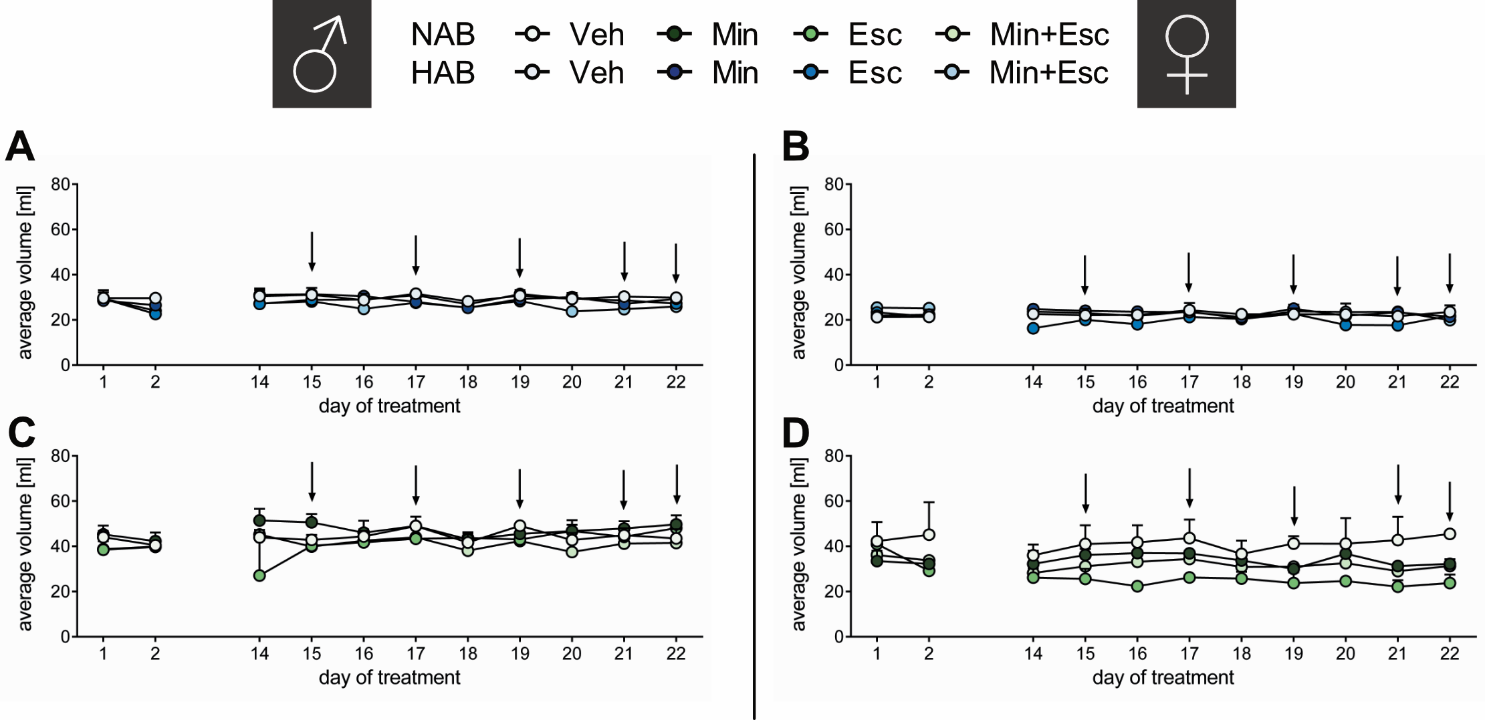
**

**Figure S1. Average fluid intake of male and female HAB and NAB rats treated with vehicle (Veh), minocycline (Min), escitalopram (Esc), or a combination of both (Min+Esc) during 22 days of treatment.** Average volume consumed by each animal per cage of male (A; n = 3-5) and female (B; n = 3-4) HAB rats as well as male (C; n = 2-4) and female (D; n = 2) NAB rats. All rats showed a stable fluid intake over 22 days of treatment independent of treatment or sex, except a slight reduction in volume on day 18. Of note, female NAB rats treated with escitalopram seemed to drink less. Arrows indicate day of experiment, i.e. the social preference day on day 15, light-dark box on day 17, elevated plus-maze on day 19, pre-swim on day 21, and forced-swim test on day 22 of treatment. Data represents mean + s.e.m.. No statistical comparison was performed due to the low sample size.

**
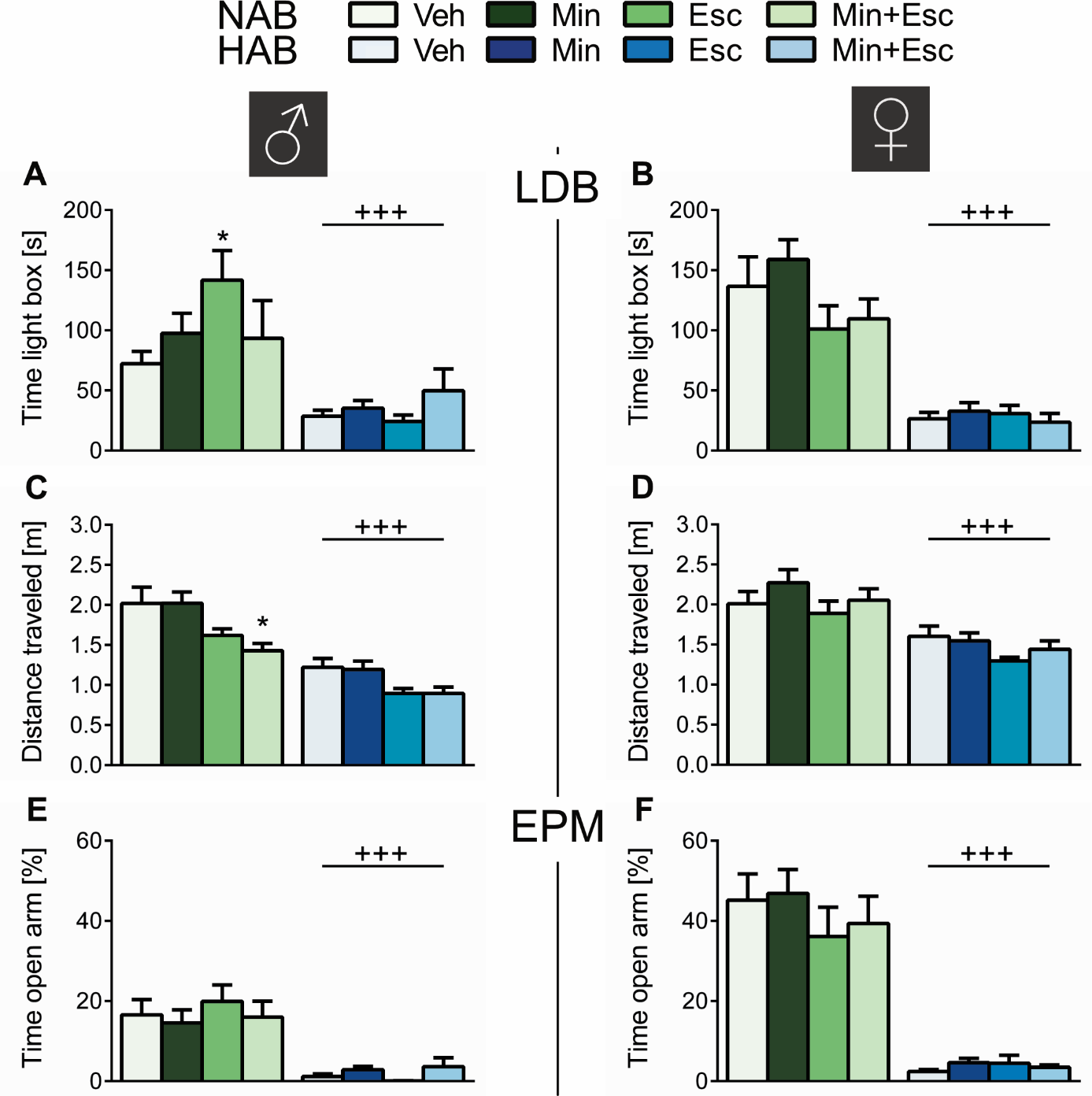
**

**Figure S2. Anxiety-like behavior and locomotion of male and female NAB and HAB rats treated with vehicle (Veh), minocycline (Min), escitalopram (Esc), or a combination of both (Min+Esc) in the light-dark box (LDB; day 17) and on the elevated plus-maze (EPM; day 19)**. Both male (A, E) and female (B, F) HAB rats showed increased anxiety-like behavior compared to NAB rats in the LDB (A, B) and on the EPM (E, F). Esc was only effective to further reduce the low anxiety level of male NAB rats in the LDB (A). Locomotor activity remained unchanged in HAB and female NAB rats (C, D) while male NAB rats showed a reduced distance traveled in response to the combinatory treatment. Data represents mean + s.e.m.; * p<0.05 vs. corresponding Veh group; +++ p<0.001 vs. NAB; Two-way ANOVA followed by a Bonferroni *post hoc* test.


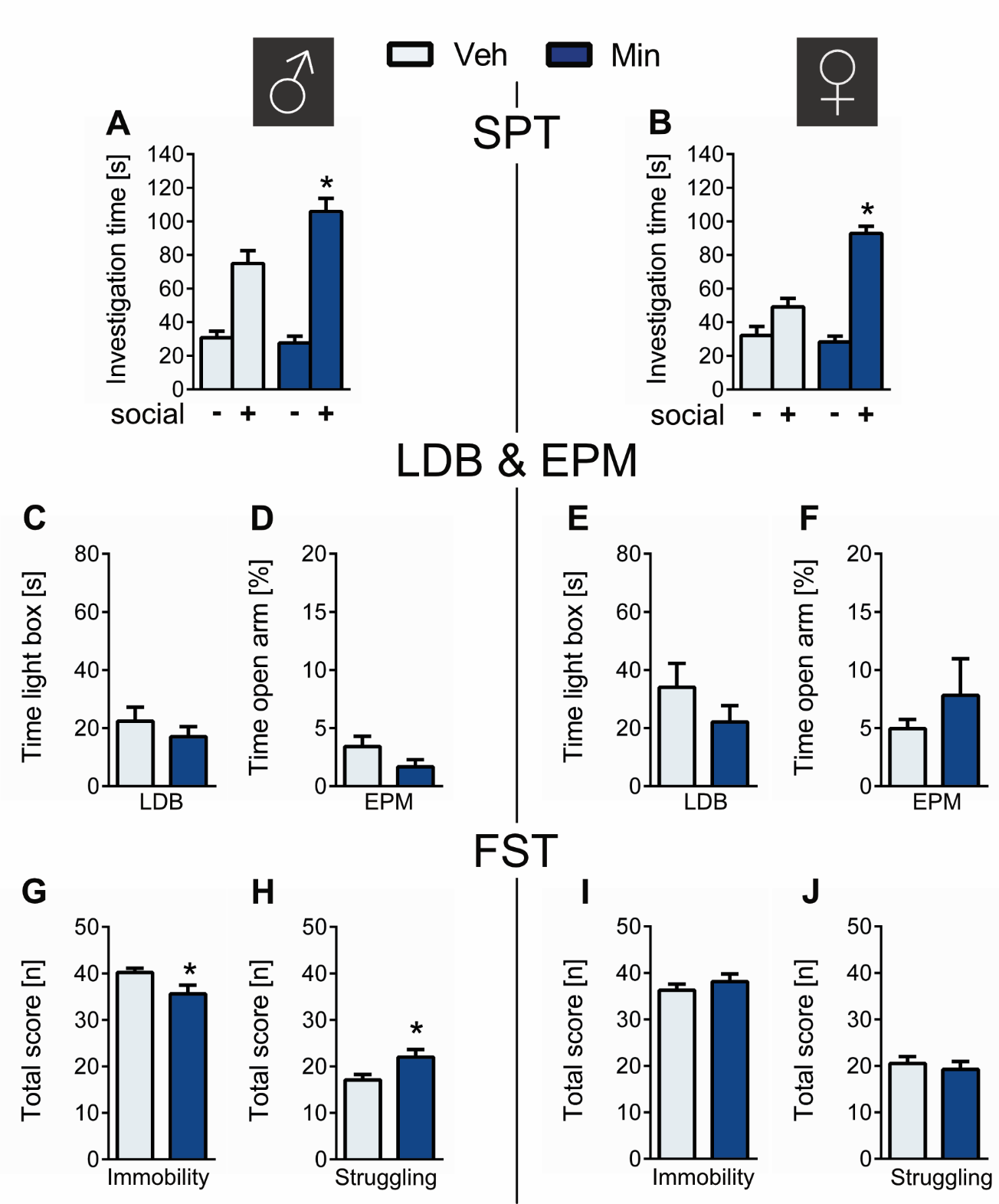


**Figure S3. Effects of 22 days of vehicle (Veh) or minocycline (Min) at a higher dose (80 mg/kg) on behavior.** Both male (A) and female (B) HAB rats showed natural social preference for a social (+) over a non-social (-) stimulus (significance not indicated) on day 15 which was facilitated by Min. Anxiety-like behavior in the light-dark box (LDB; day 17; C, E) and on the elevated plus-maze (EPM; day 19; D, F) remained unchanged. In the FST, depressive-like behavior was alleviated in male (G, H), but not female (I, J), HAB rats. Data represents mean + s.e.m.; * p<0.05 vs. corresponding Veh group. SPT: one-way ANOVA for repeated measures followed by a Bonferroni *post hoc* test; Anxiety- and depressive-like behavior: two-sided t-test.

**References**

1 Lukas M, Toth I, Reber SO, Slattery DA, Veenema AH, Neumann ID. The neuropeptide oxytocin facilitates pro-social behavior and prevents social avoidance in rats and mice. *Neuropsychopharmacology* 2011; **36**: 2159–68.

2 Henniger MSH, Ohl F, Hölter SM, Weißenbacher P, Toschi N, Lörscher P *et al.* Unconditioned anxiety and social behaviour in two rat lines selectively bred for high and low anxiety-related behaviour. *Behav Brain Res* 2000; **111**: 153–63.

3 Neumann ID, Torner L, Wigger A. Brain oxytocin: differential inhibition of neuroendocrine stress responses and anxiety-related behaviour in virgin, pregnant and lactating rats. *Neuroscience* 2000; **95**: 567–75.

4 Detke MJ, Rickels M, Lucki I. Active behaviors in the rat forced swimming test differentially produced by serotonergic and noradrenergic antidepressants. *Psychopharmacology (Berl)* 1995; **121**: 66–72.

5 Cryan JF, Valentino RJ, Lucki I. Assessing substrates underlying the behavioral effects of antidepressants using the modified rat forced swimming test. *Neurosci Biobehav Rev* 2005; **29**: 547–69.

6 Slattery D, Cryan JF. Using the rat forced swim test to assess antidepressant-like activity in rodents. *Nat Protoc* 2012; **7**: 1009–14.

7 Imase M, Watanabe K, Aoyagi H, Tanaka H. Construction of an artificial symbiotic community using a Chlorella-symbiont association as a model. *FEMS Microbiol Ecol* 2008; **63**: 273–82.

8 Lane DJ. 16S/23S rRNA sequencing. In: *Nucleic Acid Techniques in Bacterial Systematics*. John Wiley and Sons, 1991, pp 115–75.

9 Klindworth A, Pruesse E, Schweer T, Peplies J, Quast C, Horn M *et al.* Evaluation of general 16S ribosomal RNA gene PCR primers for classical and next-generation sequencing-based diversity studies. *Nucleic Acids Res* 2013; **41**: 1–11.

10 Stämmler F, Gläsner J, Hiergeist A, Holler E, Weber D, Oefner PJ *et al.* Adjusting microbiome profiles for differences in microbial load by spike-in bacteria. *Microbiome* 2016; **4**: 1–13.

11 Antón J, Oren A, Benlloch S, Rodríguez-Valera F, Amann R, Rosselló-Mora R. Salinibacter ruber gen. nov., sp. nov., a novel, extremely halophilic member of the Bacteria from saltern crystallizer ponds. *Int J Syst Evol Microbiol* 2002; **52**: 485–91.

12 Caporaso JG, Kuczynski J, Stombaugh J, Bittinger K, Bushman FD, Costello EK *et al.* QIIME allows analysis of high-throughput community sequencing data. *Nat Methods* 2010; **7**: 335–6.

13 R Core Team. R: A Language and Environment for Statistical Computing. Vienna: R Foundation for Statistical Computing. 2017.

14 Huber W, Carey VJ, Gentleman R, Anders S, Carlson M, Carvalho BS *et al.* Orchestrating high-throughput genomic analysis with Bioconductor. *Nat Methods* 2015; **12**: 115–21.

15 Quast C, Pruesse E, Yilmaz P, Gerken J, Schweer T, Yarza P *et al.* The SILVA ribosomal RNA gene database project: improved data processing and web-based tools. *Nucleic Acids Res* 2013; **41**: 590–6.

16 Edgar RC. Search and clustering orders of magnitude faster than BLAST. *Bioinformatics* 2010; **26**: 2460–1.
